# New Ways of Working to Manage and Improve Quality in Integrated Care Systems in England

**DOI:** 10.34172/ijhpm.8424

**Published:** 2025-01-15

**Authors:** Mirza Lalani, Michele Peters, Thavapriya Sugavanam, Helen Crocker, James Caiels, Harriet Hay, Sarah Gunn, Helen Hogan, Bethan Page, Ray Fitzpatrick

**Affiliations:** ^1^Department of Health Services Research and Policy, London School of Hygiene and Tropical Medicine, London, UK.; ^2^Nuffield Department of Population Health, University of Oxford, Oxford, UK.; ^3^Personal Social Service Research Unit, University of Kent, Canterbury, UK.; ^4^Picker Institute Europe, Oxford, UK.; ^5^Department of Experimental Psychology, University of Oxford, Oxford, UK.; ^6^Cicely Saunders Institute, King’s College London, London, UK.; ^7^Nuffield College, University of Oxford, Oxford, UK.

**Keywords:** Integrated Care, Care Quality, Collaboration, England

## Abstract

**Background:**

Integrated care systems (ICSs) in England were formally established in July 2022 to coordinate the planning and delivery of health and care services. A key responsibility was to address the quality of these services. Our study aimed to examine how ICSs approach this responsibility and to identify opportunities and barriers experienced in their early establishment and development.

**Methods:**

A sample of four ICSs were recruited to participate. Interviews and meeting observations were undertaken in two phases (before and after the inception of ICSs) around 12 months apart. A total of 112 interviews were carried out with senior figures in the four ICSs supplemented by observation of relevant meetings and analysis of relevant documents.

**Results:**

Regarding quality, ICSs demonstrated several new ways of working. They set-up new structures for quality governance and created whole-system strategies for quality centred on major responsibilities regarding population health and health inequalities. These strategies required new and relevant metrics to assess quality and outcomes and a greater focus upon co-production in the development of services. They aimed to strike a fine balance between long-standing requirements for quality assurance and new responsibilities for quality improvement (QI). New approaches were underpinned by new collaborations between system partners extending beyond healthcare to include Local Authorities (responsible for social care and public health) and local communities.

**Conclusion:**

To address the many challenges of quality, ICSs have created new ways of working cultivating different kinds of collaborative relationships compared to established hierarchical, siloed and top-down ways of working prior to their formation. A focus on improving population health and reducing inequalities has required a shift from "here and now" urgent problem-solving to working with longer timelines. Such changes require patience in the context of political pressure to devote efforts to more salient problems such as waiting lists.

## Introduction

**Key Messages**

**Implications for policy makers**

- The study presents early insights into the development of integrated care systems (ICSs) in England providing useful learning for policy-makers.
- ICSs provide an opportunity to address very broad and diverse dimensions of care quality such as wider inequalities, co-production and quality improvement (QI).
- In doing so, ICS will have to develop new ways of working in terms of how service quality is improved and how quality is measured as well as encouraging the development of new relationships and collaborations with local people and communities and across health and social care providers and settings.
- This will require sufficient funding and resources but also time – especially due to the current context in which health and care organisations are grappling with huge pressures in several parts of the health and care economy.
- Such pressures are likely to hinder ICSs ability to tackle population health problems and reduce inequality which poses an intriguing challenge for policy-makers and politicians in how to redress the balance in systems that are focussed primarily on treatment not prevention.

**Implications for the public**

Integrated care systems (ICSs) offer the promise of more coordinated services that reduce duplication and encourage more patient-centred care. To date, health and social care services have defined the quality of care provided by services in terms of its effectiveness (how well it works), its safety (minimises harm) and how positive (or not) the experience of care is among patient and users. The goals of ICSs go further, offering new ways of thinking and working to improve the quality of health and social care. This includes a greater focus on preventing ill health and reducing the inequalities that exist in health among different population groups. To deliver these goals, ICSs will have to build new relationships with patients and users of care, local people and communities as well as between the different health and care organisations within a system.

Healthcare systems in many countries are now focused on integration of services evolving from core organisations such as hospitals that traditionally operated independently of each other. The reasons for the trend toward integration are agreed. Populations are ageing, so that increased proportions of users must cope with frailty, complex diseases of ageing and multiple morbidities.^1^ This profile of health problems requires that patients are managed by increasingly complex care pathways across diverse services and organisations. The term “integration” refers to a “coordination” of traditional silos of care across (horizontal) and within (vertical) systems, organisations, services, and service providers.^2^

 The English National Health Service (NHS) has attempted to facilitate the integration of services by system-level changes. Initial pilot approaches, Integrated Care Pilots, followed by Integrated Care and Support Pioneers were small in scale and attracted only modest central funding.^3^ In 2015, a number of “Vanguard” sites were identified and provided with more substantial national funding and support to carry out local service innovations that would reduce barriers between the various sectors of health and social care.^4^ Lessons from these pilots informed the Five Year NHS plan^5^ for the service as whole, a key goal of which was improved integration. In the following year, multiple local Sustainability and Transformation Partnerships were established, again with central support and funds to facilitate greater collaboration in delivery between NHS organisations, but also more partnership working with Local Authorities. In England, Local Authorities are local government structures that are responsible for public service provision including social care and public health^6^ but are independent of the NHS. To date, evaluative evidence of integrated care initiatives both nationally and internationally has indicated only limited evidence of success and varying views of what constitutes integration.^7^

 Most recently, the English Health and Care Act 2022 established integrated care systems (ICSs) which are funded centrally by the UK government’s Department of Health and Social Care. ICSs comprise statutory integrated care boards (ICBs) and integrated care partnerships (ICPs) in each of 42 local system areas of England.^8^ Formally established in July 2022, the main mission of ICSs was focused on improving outcomes in population health and healthcare and tackling inequalities in outcomes, experience and access.

 Quality quickly became a central focus for the newly established ICSs. The National Quality Board (NQB) was previously established by NHS England (NHSE) to provide authoritative guidance to the NHS on all matters relating to quality. In the early evolution of ICSs, NQB guidance was influential on emerging structures and processes.^9^ Its breadth can be illustrated by the emphasis on population health, aiming to co-produce future services with the public and use of transparent measures of quality and safety all of which are new ways of working for health and care organisations.

 Quality in terms of improved health outcomes, safety and experience is a main driver in most initiatives to increase coordination and integration of services. Much of the evidence base on the impact of integration upon the quality and outcomes of services is micro-focused, that is to say, drawing on evaluations of service innovations in specific services or patient groups, for example innovations to implement integration in a local diabetes care service.^10^ To understand the possible impact of ICSs on the quality of services requires a system-level approach to match the level at which they are likely to operate, at least in their early years.

 Through this study we aim to understand how ICSs approach their responsibility to improve quality and to identify opportunities and barriers experienced in their early establishment and development. Hence, this study aims to assess how ICS are employing new ways of working to assure and improve the quality of care.

## Methods

###  Study Design

 The study used qualitative methods comprising two phases of semi-structured interviews and meetings observations conducted between November 2021 and May 2022 (before the inception of ICSs) and between January 2023 and May 2023 (6 months after ICSs launched).

###  Study Setting

 We purposively selected four ICSs for participation ensuring variation in terms of geography (urban/rural), demography (population size and diversity), and pre-existing system architecture such as length of history as an ICS or related experiences eg, Sustainability and Transformation Partnerships. In selecting the ICSs we were keen on identifying the commonalities among them rather than making specific comparisons about the progress of an individual ICS. Where notable differences exist with regard to the approach to quality we have highlighted them.

 A brief overview of the constituent organisations within each ICS can be found in Table 1.

**Table 1:** Participating Integrated Care Systems

**ICS**	**Organisations**
A (population 1 million)	Four place*-based partnerships with 4 Local Authorities, 3 Community and Mental Health Trusts, 2 Acute Trusts, 2 Ambulance Service Trusts, 102 GP practices, and 4 Healthwatch**
B (population 1.8 million)	Five place-based partnerships with 4 Local Authorities, 1 Community and Mental Health Trust, 4 Acute Trusts, 1 Ambulance Service Trust, 248 GP practices, and 4 Healthwatch
C (population 1.1 million)	Five place-based partnerships boards: with 2 Local Authorities (county councils), 3 Acute Trusts, 3 Community and Mental Health Trusts, 1 Ambulance Service Trust, 105 GP practices, and 2 Healthwatch
D (population 2 million)	Seven place-based partnerships with 8 Local Authorities, 2 Community and Mental Health Trusts, 3 Acute Trusts, 276 GP practices, and 8 Healthwatch

Abbreviations: ICS, integrated care system; GP, general practitioner. *A “place” is a geographical level within an ICS structure which comprises a population between 250 000-500 000. ** Healthwatch are a place-based organisation that collect patient and user feedback on their experiences of using health and social care services.

###  Data Collection and Analysis 

 The primary method of data collection was qualitative interviews, supplemented by meeting observations and documentary review of relevant meeting papers and strategy documents (See Table 2). A total of 112 interviews were held with senior leaders and other key stakeholders across the four ICSs – 70 in phase 1 and a further 42 in phase 2, with 14 respondents interviewed in both phases. We purposively sampled a diverse range of actors from across the health, social and voluntary sectors as well as those representing local people and communities to participate in interviews. This ensured that we garnered a broad range of perspectives on the management and assurance of quality in ICS.

**Table 2:** Overview of Research Methods

**Research Method**	**Data Sources **
Interviews (n = 112, 14 interviewees were interviewed twice, 12-18 months apart)	ICS Executive team, ICB and ICP members, other ICS staff, CCG representatives, place-based staff (n = 45): CEO, independent chair, chief information officer, medical director, other executive, and non-executive members CCG staff: Clinical chairs, CCG directors: transformation, performance, assurance Chief medical officer, chief nurse, quality leads, lead for quality development, and clinical quality manager Place based directors Senior representatives in health providers NHS acute, CMHT, and primary care (n = 33): CEO/Deputy CEO, director of nursing, other lead roles eg, chief quality officer, Trust quality leads Local Authority representatives: Director of Adult and Children Services, Director of Public Health (n = 19) Healthwatch, VCSE, and other public involvement representatives (n = 15)
Observation of meetings	Meetings included: Integrated Care Partnership Board, Integrated Care Board, CCG Governing Board, Quality forum, System Quality Group, System Delivery Group, and ICS CEO group
Documentary review	Several documents pertaining to ICS and quality including ICS strategic plans, ICS quality strategy, and framework

Abbreviations: ICS, integrated care system; ICB, integrated care board; ICP, integrated care partnership; CCG, Clinical Commissioning Group; CEO, chief executive officer; NHS, National Health Service; CMHT, Community and Mental Health Trust; VCSE, voluntary, community and social enterprise.

 In both phases, interviews were guided by a topic guide. In phase 1, we focussed on the overall ICS perspective on quality, how the ICS is organised to address quality, the internal and external influences on the ICS’s approach to quality, challenges faced and the capacity of the ICS to address quality. Our focus in phase 2 was facilitated by an interim report of phase 1 findings, a related webinar on our observations from the first phase which we held with quality leads from the four ICSs and a review of relevant ICS documents. In follow-up discussions with these leads we agreed key topics jointly considered to be central. We covered aspects such as progress on ICS and quality since phase 1, relevance of the Quality Management Systems framework,^11^ progress on quality improvement (QI) approaches, metrics, partnership working and strategies for population health/inequalities and co-production.

 All interviews were conducted on Microsoft Teams, audio-recorded and transcribed verbatim. Written informed consent was sought prior to interview. Interviews lasted between 45-60 minutes. We conducted a thematic framework analysis, identifying patterns and themes in the data.^12^ The qualitative data management tool NVivo (version 12) was used to manage and code the interview data. The research team met at frequent intervals during data collection and analysis to discuss the development of a coding framework and to identify themes in the data. The coding framework was updated iteratively. For phase 1 we developed a highly detailed coding framework using deductive and inductive approaches which also formed the basis of our coding in phase 2 with a few adaptations where necessary. For both phases, the analysis was informed by relevant insights from the review of ICS documents, the academic literature on quality in care and ICSs as well as organisational literature on managing change, complex decision-making processes and policy implementation.

## Results

 Our data revealed three key themes: setting up ICS and quality mechanisms, dimensions of quality and developing quality by informal means. We also identified an underpinning and cross-cutting theme of “new ways of working” indicative of the broader approach to quality employed by ICSs.

###  Setting up ICSs and Quality Mechanisms

 The scale and scope of work involved in all four ICSs to set up structures and processes to govern the new system was extensive. The previous system built around Clinical Commissioning Groups (CCGs) (statutory NHS bodies responsible for the planning and commissioning of healthcare services for their local area) had to be restructured and absorbed into the new system; with new mechanisms needing to be put into place for decision-making, finances, and commissioning. This restructure was highly labour intensive and complex in a context of considerable challenges. Influenced by several external bodies, guidelines and frameworks, the ICSs needed to be strategic and prioritise how they tackled the re-structure. Hence, this theme is further categorised into the overall context within which ICSs were instituted, external guidance (from government agencies), setting up the system and place structures and setting up quality mechanisms.

####  The Context

 The context within which these new structures needed to be created was one of multiple, overlapping challenges for the health and care system, and the staff, many of which were exacerbated by the COVID-19 pandemic. Together with additional factors such as winter pressures, there were long waiting lists across the majority of NHS services (eg, elective care), staff challenges (eg, burnout, workforce shortage, high turnover, and redundancies and strikes); widening of health inequalities; and financial challenges including needing to cut administrative budgets by 30%. All these impacted on the quality of services and progress with developing the ICS structure.

 Additional challenges arose due to the misalignment of jurisdictional boundaries between Local Authorities and ICSs creating fragmentation and hindering seamless service provision. Local Authorities operate within predefined geographical limits, while healthcare often transcends these boundaries. Participants reported that diverse political and administrative structures, with varying priorities and conflicting policies, could impede the harmonisation of efforts. Differing accountability mechanisms between Local Authorities and the NHS (within one ICS) could lead to ambiguity and challenges in decision-making, resulting in a need to balance local democratic accountability with system-wide decision-making. Coordinating decision-making processes and achieving consensus among this multiplicity of accountability structures could be a delicate task. Despite the challenges, there was a strong commitment to working together across the ICS.

 “*When [individual] arrived he said, well hold on a minute, why are we doing it without the co- terminosity ? Let’s have a real signal to the whole of the ICB that we’re going to do it in a way that promotes engagement with the local authorities. So it’s an enabler, it helps the local authorities understand that they are important, and it sends a message to those working in health that engagement in social care is a key thing going forward” *(ICS B, Medical Director).

####  External Guidance

 Many of the key components of ICSs, including their approach to quality, are externally prescribed by regulators or arms-length bodies. The Care Quality Commission, a key regulator of the English NHS and social care services, was often highlighted as influential in terms of quality or assessing system performance. While there were no Care Quality Commission inspections of the ICSs at the time of our study (but introduced shortly afterwards), they were considered as a potential catalyst for driving improvement forward. Equally, a range of national guidance, policies, strategies or frameworks such as the NHS Long-term plan and NQB guidance to name but a few,^10^ were highlighted as influencing ICS planning. Participants acknowledged that external guidance helps with standardisation across ICSs. However, they also perceived the guidance as often not fit for purpose, too prescriptive and lacking salience in the context of local needs. Hence, some developed their own approaches whilst trying to keep these balanced with the national guidance.

 “*…we will have national and system drivers that will be telling us what we need to focus on in terms of quality. But I equally feel that there should be a bottom-up approach, you know, what is our place telling us about quality? What are our population telling us about quality? …. It’s the National Quality Board that are really being clear that this is about experience, access, outcomes. So, it would be remiss, of course, not to be focusing on those things, but then we’ve got to do or our job, what does that mean for us, locally?” *(ICS B, Quality Lead).

####  Setting up System and Place Structures

 At baseline, old structures were being re-organised eg, CCGs merged and new commissioning structures set up ie, the formation of the ICB (absorbing the merged CCGs) along with ICPs established in parallel. Participants were mostly positive about their progress with the re-structure seen as an “exciting” opportunity, albeit challenging and time consuming. However, a small number of participants were less convinced by the progress and questioned the effects that integration would have on patient outcomes. Across the four ICSs we observed some gradual progress in place-based (local borough) planning, structures and mechanisms between baseline and follow up. That said, the pace of development across the ICSs varied with the need to tailor approaches to the local population.

 “*We’ve got four places, which have got four very different characteristics in terms of population, so you can’t really sort of just assume that something here should be exactly the same somewhere else”* (ICS A, Director of Strategy and Transformation).

####  Setting up Quality Mechanisms

 The NQB published several documents to support the development of quality in ICSs which specified an agreed definition of quality and a vision for how quality can be delivered in the ICSs (2021) followed by guidance on System Quality Groups (2022). These guidelines meant that quality structures within the ICSs were similar. At system level, the primary responsibility for quality was usually with the Chief Nurse or Director of Nursing. Some ICSs had quality leads for each of their places.

 In terms of strategy, some ICSs developed a separate strategy for quality, whereas others embedded quality within their overall strategy. Strategies were developed collaboratively with the involvement of multiple partners. All ICSs had ambitious visions for quality and although expressed in different ways, the strategies focussed on: (*i*) covering the entire lifespan of their population; (*ii*) population health and inequalities; (*iii*) staff wellbeing; and (*iv*) providing high quality and efficient services. Clinical priority areas included: long-term conditions, mental health, cancer, and maternity services.

 “*For the quality strategy we took a much more inclusive way of working. I led some workshops and brought all the different members of our system together to talk about a strategy that would cover not only health but the Local Authority, the voluntary sector, all the different touchpoints in a person’s life. So we’ve done that in an inclusive way… its not just expecting it to be the responsibility of one, its everybody’s responsibility” *(ICS C, Executive Director of Nursing).

 All ICSs worked towards developing a whole system approach for quality using either Juran’s Trilogy of Improvement encompassing quality planning, quality control and QI^13^ or the Quality Management Systems approach which has evolved from Juran to also include quality assurance. All ICSs had a strong focus on QI across the whole system, with system leaders repeatedly stressing the need to move away from assurance towards improvement. While most system leaders were optimistic with their new approach to quality, some were concerned that it may not be actionable due to contextual challenges.

 As per the NQB’s guidance, two committees were set up for quality: the ICB Quality Committee and the System Quality Group. The Quality Committee was a ICB sub-committee with an assurance function (ie, to monitor routine NHS performance data for outliers). Meeting observations revealed that most attendees were from the NHS and meetings predominantly focussed on NHS risks and key performance indicators such as ambulance waiting times and bed capacity. The System Quality Groups were designed as a strategic forum to facilitate engagement, intelligence-sharing, learning, and QI across the ICS. Membership included ICB quality leaders and representatives from all partner organisations including local authority and patient representatives. While these groups were still in development even at the follow up stage, their main goal appeared to be to facilitate system level learning.

###  Dimensions of Quality 

 We identified four key dynamics shaping quality: (*i*) tensions between quality assurance and improvement; (*ii*) improving population health; (*iii*) the approach to measuring quality and performance (metrics and measurement); and (*iv*) involving people in enhancing the quality of services (co-production). These are discussed below.

####  Quality Assurance and Improvement 

 Overall ICSs are developing approaches to support systems, place and provider improvement with a focus on inequalities but there were concerns that progress could be hindered by an assurance-centred mindset at both the ICS and national level. NHSE’s approach to quality in ICSs was seen as “report heavy” with top-down accountability and a focus on areas that may pose a risk to patient safety – often determined by the political agenda eg, problems in prompt access to primary care services. Several interviewees remarked on national requirements for assurance including targets, metrics and reporting which perpetuated an established focus on performance particularly among ex-CCG staff. Many wanted to see a reduction in the quantity of reports but ex-CCG staff, in particular, tended to be reassured by reporting, as they felt it supported risk management and assured system safety.

 The presence of ex-CCG staff in the ICB had resulted in the retention of a contracts-based management approach. Hence, at the system level, quality was equated with meeting performance targets and assurance was viewed as being the primary driver in prioritisation for resource allocation. However, other system leaders felt that there should be much less preoccupation with secondary care performance metrics such as reducing Accident & Emergency and elective surgery wait times which were not necessarily helpful when pursuing wider outcomes for an entire ICS. There were concerns that expediting patients through a service was a greater priority for some system leaders than the quality experienced by patients while under the care of a provider.

 With these issues in mind, several interviewees suggested the need for a collective mindset shift to less assurance-focused ways working. However, it was recognised that, despite a key benefit of ICSs being the removal of the purchaser/provider split and greater attention paid to improvement, ICBs were still, in effect, commissioners – accountable for performance which may lead to a return to assurance when systems were under pressure.

 “*The commissioner/provider split, that’s gone out the window, but yet we’ll still be employed by a commissioning body. So if we’re not commissioners, what are we? … You want us to behave differently … but you still want us to provide assurance. How do we behave differently when you haven’t clarified the range, the remit, the scope for us to operate at place … We’re ambitious to do it, we are willing and want to learn … and break out of so much assurance burden and perhaps do something more creative in improvement world” *(ICS B, Director of Nursing and Quality Lead).

####  Shifting to Focus on Quality Improvement

 ICSs were keen on embedding a culture of improvement throughout the system such that QI becomes normalised in everyday work. This would require enhanced collaborative working across systems, within boards and teams and greater leadership in terms of knowledge and expertise and the ability to engage and motivate others. For example, some described QI supporting staff to feel joy in their work, improved psychological safety, greater openness and transparency ie, the relational elements of QI that are seldom recognised. Individuals in senior roles who had QI expertise were seen as being able to support diffusion of QI ways of working across the system. One example was an approach to discharge in one acute Trust which has involved a joint system improvement exercise involving community care, social care and voluntary care.

 “…*we had a discharge workshop because that was a prime example of where we could bring council and third sector, our trust and the community teams together around one specific area. I worked with the continuous improvement team with [Trust] to say, right let’s have a different lens on it. Let’s not look at you getting people out of the hospital … Let’s look at how we can look at the issues together as a system and then use some of your techniques to understand that” *(ICS D, Director of Place Based Partnerships).

 QI work in some ICSs centred primarily on acute Trusts. Some believed that improvement approaches ought to be enacted earlier in the care pathway in primary and/or community care which could be achieved through working on wider themes such as pathways for cancer care which in turn would demonstrate salience to wider groups of providers.

 Participants suggested that a system focussed approach to QI would require building a QI infrastructure such as developing improvement training hubs to build capacity as well as bringing together several disparate programmes, leveraging the collective expertise and strengths of individual providers. For example, one ICS was considering bringing in subject matter experts from the Institute of Healthcare Improvement. It was also suggested that innovation should be driven in partnership with universities supporting research/evaluation and a more evidence-based approach.

####  Improving Population Health

 Population health, including the reduction of health inequalities was a core aim for ICSs. Indeed, we noted a gradual shift away from describing quality in terms of its traditional (clinical) domains—safety, experience, and effectiveness to it being considered in terms of population health and inequalities with a focus on equity and access. Participants also described a desire to move towards prevention to try and identify those at risk of deterioration, which would require greater financial investment and efforts in upstream interventions in the community. ICSs have started to invest accordingly, with population health leads and teams being recruited at place. While systems recognised that seeking to reduce health inequalities is *“the right thing to do,”* there was some acknowledgement of the challenges in doing so in systems under tremendous pressures, financial and otherwise.

 To help reduce health inequalities, NHSE have developed Core20PLUS5,^14^ a set of approaches, that set out five areas of clinical focus for improvement among the most deprived 20% of the population, plus additional populations identified at a local level. Broadly, ICSs considered the strategy a helpful framework as it provides a clear starting point on which work around inequalities can be built, while still having the flexibility to be shaped to address local population needs. It had the added benefit of raising awareness of health inequalities among clinicians and more generally stimulating ICSs to develop a culture of looking at care provision with an inequalities lens while also supporting partnership working towards addressing inequalities. That said, there was some scepticism about the relevance of Core20PLUS5 to Local Authorities with much broader, non-clinical perspectives on inequalities in health. Some participants questioned the remit of the ICS and whether ICSs should be leading the development of strategies to address the wider determinants of health. Non-health stakeholders suggested that addressing inequalities must be a collective endeavour with Local Authorities to avoid the risk of duplicating functions particularly in sectors within which ICSs had little experience such as housing.

 “*So I think it’s not necessarily helpful for there to be at scale discussions about housing for example. I mean housing may be of very significant relevance and concern to the ICS, but actually I think it probably makes sense for them to say housing is really important but housing, is best discussed at place” *(ICS A, Director of Public Health).

####  Metrics and Measurement

 Emphasis is shifting away from a focus on performance indicators to identifying suitable metrics to capture the multiple dimensions of quality and present these in a form that can drive decision-making. Multiple health-orientated indicators already exist for service and provider-level assurance, but there remained a relative lack of information on wider determinants of health coming from social care and public health and a means of linking these with health data. ICS also faced challenges capturing quality across care pathways and measuring the impact of integration. There is an appetite to move to more outcome-based measurement and see the development of person-centred outcomes. Some interviewees acknowledged the limitations of quantitative data alone to provide a rounded view of quality and advocated for wider use of local insights derived from qualitative data collected from patients/service users and staff associated with their experiences of delivering and receiving care.

 Barriers thought to inhibit access to and utility of data to support quality in ICS included slow progress in establishing data sharing agreements and the disparate availability of integrated care records (a consolidated patient/user record identifying the care provided by different services).

####  Co-production

 Co-production was considered as a potentially key mechanism in improving the quality of services. Although there is some evidence of a good understanding of its principles across the ICSs and recognition of its potential importance, its development is in its infancy. Co-production appears driven top-down from NHSE guidance on the need to develop strategies in partnership with patient, public and community voices. There was emphasis on “engaging users,” “listening” or “hearing the patient’s voice” but little was discussed in terms of how such insights and experiences would be used to co-produce solutions or to influence improvements in quality which created some disparity in the equality of partnerships.

 “*I have a level of scepticism when people start talking about co-production because we talk about … when we will introduce co-production to this project … we should have had it there right at the beginning. We spend a lot of time talking about co-production, what it looks like, what it means … but we’re not there yet. I don’t think we’re radical enough with it” *(ICS D, Director of Nursing).

 One of the main challenges identified to embedding co-production were ensuring that the user and community voices were representative particularly when local communities were made up of diverse populations. There remained a widespread reliance on Healthwatch to represent the patient/user voice even though many leaders acknowledged this provided only a limited view which was not representative of the wider community. It was widely acknowledged that co-production worked best at place level working with specific populations rather than at system level. Crucially, learning needed to happen from sharing good examples and positive change to ensure co-production was not a tokenistic, tick box exercise. Positive examples of co-production practice that were cited across ICSs were for specific services (eg, maternity), settings (eg, local authorities) or populations (eg, LTBTQ+ [lesbian, gay, bisexual, transgender, queer, and additional identities]).

###  Quality by Informal Means

 We have described several formal approaches to managing quality, however, the interviewees also highlighted informal means by which quality was being managed, centred primarily on developing and cultivating relationships with other provider organisations from across the ICS. This is explored further below under the sub-themes of establishing relationships and relationships as a driver for improving quality.

####  Establishing Relationships

 As new entities, ICSs largely appointed new leadership. They were required to work with a wide array of organisations within the NHS and beyond. They had to build new partnerships across organisational boundaries with entities developing their own agenda and ways of working in the new environment. Within the NHS, the powerful acute Trusts were reforming into provider collaboratives (partnerships that bring together two or more NHS Trusts to work together at scale to the benefit of their local populations). Primary care had lost some of its influence via CCGs and was being re-configured into primary care networks. To impact on quality systems, leaders acknowledged the centrality of establishing relationships with frontline services at place level. Developing partnerships was dependent on building relationships and trust between unfamiliar partners.

 “*I think, you know, looking at the quality aspects of doing that in an ICB is absolutely fundamental to getting to that result, because if you don’t think about quality very differently, you’ll just think about it from, the target driven, organisational, siloed approach as opposed to how do we collaborate – you know, collaboration is absolutely essential to all of this with all partners” *(ICS B, ICB Non-executive Member).

 Some ICSs had initially been at an advantage in terms of such informal processes, having historically stronger relationships across their footprint, especially where they had been early adopters of place-based collaborations or Sustainability and Transformation Partnerships. Similarly, new relationships had been forged through the COVID-19 pandemic when closer working between health, social care and community providers had been imperative. However, many recognised the challenge of reaching beyond traditional partners and into place and frontline services. The disruption generated by boundary reorganisation, setting up ICS governance structures and staff turnover as a result of the merging and then disbanding of CCGs compounded this issue. In general, there was a sense that some progress had been made in strengthening relationships across health and non-health partners in the year since ICS formation. Now that governance structures were, for the most part, in place and key roles filled, ICS quality leaders felt there was an opportunity to increase the pace of collaborative work on quality.

 However, there was scepticism, particularly amongst non-health partners, as to the likelihood of equal partnership in this endeavour. Local Authorities leaders described how engagement felt a “one way” mechanism for the NHS to address pressing issues such as delayed discharges. They also highlighted the lack of understanding within the NHS of their contrasting structures, governance and cultures and the impacts this has on their ability to engage in decision-making on behalf of their own organisation or represent others. Smaller organisations from the voluntary and community sector felt that there needed to be more appreciation of their capacity to engage in the work of the ICS and their support needs addressed so that they could step up to this role. The fact that NHS partners dominated agenda setting and framed issues in health-centred, technocratic language added to the sense of unequal partnership amongst non-NHS leaders. Hence, relationships between the NHS and Local Authorities over matters of quality were sometimes strained because of what was seen as undue emphasis on clinical frames of reference.

 “*I think there’s also terminology and language is really important. One of the things I always say, when documents arrive is, you’re saying system partners but all over this document it says clinical. Social care is not clinical. In fact, we’ve taken forty years to de-medicalise social care and we’re not going back. We understand fully the need for clinical governance, but I think explaining clinical and care are two different areas” *(ICS B, ICB Non-executive Member).

####  Relationships as a Driver for Improving Quality

 As partners cannot mandate action in a different organisation ie, the NHS cannot mandate action from a Local Authority, ICS leaders recognised that they need the need to establish mechanisms for collaborative accountability for quality. This was dependent on having the freedom to shift relationships from transactional, service target-based to supportive and focused on patient-centred outcomes. Some interviewees suggested that new ways of addressing quality shortfalls would be best developed through deepening understanding of partner organisations’ issues, working alongside partners to solve these issues whilst using a different set of soft skills, language, and conversations. Quality leaders recognised the importance of embedding members of the ICS quality team at place and close to providers to facilitate communication of this cultural shift. They hoped that strengthening trust across the system would create an environment where openness and transparency helped identify issues earlier and encouraged partnership working to establish the best approach to address them.

 Also tacit in the outgoing system was a search for fault and blame, the need to identify a specific organisational source for every identified problem resulting in enquiries and investigations to address organisational failing. Respondents commonly noted the contrast in ethos in the newly established ICSs dominated by collaborative styles of working. New forms of relationship were seen as “respectful” and working together to reach shared solutions. Respondents commonly described their work as the ICS dealt with other organisations as involving “conversations” and “posing questions,” in mutual non-adversarial relations.

 “*And then we pose questions to everybody, particularly our providers. Is this an approach that you see meriting for you to adopt, for you to be able to do the same. And it’s posed as questions, as opposed to ‘we want you to do this.’ And we also ask what more do you think you could do? What could you do differently? What else can you do? It’s a series of questions around improvement for those providers to adopt” *(ICS D, Chief Nurse).

 As well as a different style of interacting, ICSs involved different ways of thinking about problems, precisely because of their system-level remit. Problems arising in health and care are not often due to one organisation’s failings and require instead a systemic focus. As well as diagnosing system-level solutions to service problems, the requirement to take responsibility for population health required more complex ways of thinking and analysis. The need to identify up-stream causes of ill-health and even more so, interventions to address causes were seen as conceptually stimulating, vitally important but especially challenging. Most obviously, respondents frequently described the need for providers and their commissioners to focus on the “here and now,” to address the many pressing, urgent services in their remit.

## Discussion

 Our study aims to provide insights about the early development of ICS through the lens of quality. Prominent among these has been new ways of working in ICSs. This is apparent in the development of new governance structures as well as in the vision of senior leaders, shifting the focus from commissioning to collaboration and relationship building based on trust. While an assurance mindset remains both at the national level and locally among some stakeholders, ICSs are keen to adopt an improvement culture which will be crucial to improving population health, reducing inequalities and instituting prevention focussed services. However, this approach is likely to be tempered by existential pressures on the health and care system.

 There are several examples in our data of the new ways of working in ICSs through the lens of quality. Notably, ICS have been charged with an extremely broad and inclusive definition of quality. For some time, the NHS worked with an approach consistent with widely accepted clinical perspectives to quality: safety, effectiveness and (patient) experience.^15^ Similarly, these three dimensions are incorporated into the approach to care quality from the social care perspective with an additional focus on ensuring services are equitable and person-centred.^16^ However, the new ICS remit goes further, encompassing access and especially inequalities in access to services with the goal of improving population health. There has long been recognition that the NHS has a role in addressing health inequalities,^17^ albeit with ambiguities about responsibilities for wider social determinants. Seldom have specific bodies been charged so clearly, directly and firmly with addressing health inequalities as have the ICSs. The Core20PLUS5 programme is somewhat emblematic of this directive approach to inequalities.^14^ The challenging nature of such a broad approach to quality is the remit to make a noticeable and significant improvement across such disparate aspects of quality, given likely varieties of mechanisms of intervention within and beyond services.

 Our findings indicate a recognition of the importance of a broader approach to quality across the participating ICSs and the organisations within them. However, the timing of legal set-up of ICSs created considerable distractions from their quality agenda: post–COVID-19 recovery pressures, workforce strikes and wider, longstanding, workforce issues of poor of morale and staff shortages all created major headwinds on progressing quality. Going forward, there are several challenges in implementing a broad quality strategy. ICSs inherited a managerial system and workforce accustomed to commissioning mechanisms focused on quality assurance, rather than improvement. Voluminous data supported this commissioning function and new metrics and approaches to measurement were seen by respondents as essential with a focus on outcomes and experiences to inform the quality agenda. The ambition of ICSs is to create local level metrics for use in system dashboards that would identify problems in services as they arise are being impeded by the longstanding barriers of information governance, a lack of data sharing agreements and data availability.^3,18^

 As already implied, within ICSs, the NHS must work at establishing new relationships with a wide range of other, independent organisations to pursue their responsibilities for quality. This is most obviously the case in working with Local Authorities, responsible for social care and public health but also other creators of good health such as housing and education. Local Authorities are completely independent from the NHS, accountable instead to a local electorate. Much energy and effort in the start-up phase of ICSs had to be devoted almost exclusively to establishing informal relationships and trust as the mechanism for partnership working. At this early stage of development, an informal collaborative way of identifying and addressing quality issues was seen as dependent on relationships. A year in, NHS trusts were forming provider collaboratives with their own agreements, logic and powerful influences; the main route to influence over such bodies by ICSs was informal. ICSs at the top of the administrative chart also had to make considerable effort to reach down to impact on quality of delivery at the local, place level with few or no hierarchical levers and only some jointly appointed posts and still evolving relationships to draw on.

 The ways of working noted here are consistent with other research on ICSs that stresses the centrality of collaborative and trust-based working.^19^ Sanderson et al focus on a slightly earlier period in the establishment of ICSs when very considerable energy was invested in reaching clear and transparent agreement in governance between partners. The same efforts were observed in our ICSs in reaching agreement on how to manage quality. Sanderson et al draw on the work of Ostrom to argue that informal collaborative organisations are fragile if not based on elaborated agreement on the rules of the game.^20^ As in our study, they found that ICS staff valued informal and collaborative ways of working compared to traditional vertical accountability. A study of Sustainability and Transformation Partnerships, the precursor of ICSs, noted a tension between traditional hierarchical forms of authority in the NHS with the informal, relational networks required to facilitate successful working in Sustainability and Transformation Partnerships.^21^

 Indeed, a shift was observed even within the approach to commissioning moving away from a bureaucratic mode to one centred on partnership. “Conversations” appear to be the new mode of operation, invariably preferred over the formalities of the commissioner–provider split. Conversations facilitated more productive consideration of how jointly to solve problems. Quality problems were very often seen as system-based, due to a range of factors operating across a range of organisations rather than due to a single provider. Most crucially conversations to reach shared solutions avoided what was seen as the old style of commissioning in which the goal of interactions was to find an organisation to “blame” for quality failings; once “blamed,” an inquiry ensued to fix that organisation’s failings.^22^ The new approach employed in ICSs encourages a move away from the endless ritual of monitoring of targets and focuses more on improving key outcomes for patients that can be identified through co-production and more broadly engaging with local people and communities.

 Another significant finding of our work is the sheer scale of ambition of ICS. This shift to a whole systems integration may have been gradual and incremental over the last decade but it still represents a seismic shift in terms of structure, governance and strategic intent. There are parallels to the past eg, the establishment of Regional and Area Health Authorities in the 1970s brought health and public health together but had no direct responsibility for social care.^23^ Internationally, evidence of proven benefits to populations of integration of whole services is modest; the example of Accountable Care Organisations is the nearest instance of an organisation established to provide overarching management of services to a defined population; the evidence of effects of Accountable Care Organisations on quality and outcomes is complex and unclear.^24^ Similarly, the responsibilities for quality placed with ICSs are unprecedented in their range, scale and visibility. The evidence base for interventions to reduce, rather than simply describe, inequalities in health and improve quality and outcomes of services is complex and identifies no quick or simple fixes.^25,26^ The analytical and organisational skills to deliver such improvements at the scale of a system are daunting, as indeed are the efforts that would be needed substantially to co-produce services. As also argued by Sanderson et al,^19^ to this array of expertise, needs to be added the softer but equally important negotiating and relational skills required of ICSs. The relational elements of integration such as communication, professional cultures, building trust and partnership working are recognised as enablers for multi-disciplinary and multi-sectoral care at the operational and service delivery level^27^; our findings also signal their significance at the system level.

 A range of external bodies are being developed to support ICSs in fulfilling their duties, in areas such as QI and population health.^8^ However, ICS respondents often expressed mixed feelings about the role of external bodies such as NHSE, partly because of resulting ambiguities in the division of labour between bodies. Previous research has highlighted potential problems when third party bodies such as NHSE effectively offer “meta-governance,” that is, policies and implementation plans that mediate between government and providers.^28^ ICSs may be heading toward occupying similar and potentially competing space in governance terms, with further risks of confusion of roles. In any case, the availability of external advice to ICSs does not necessarily solve inherently complex challenges of how to improve quality in the local context.

 A key strength of our work was the breadth and diversity of the study participants in terms of their roles and the organisations they represented. A limitation to our study was that participants were senior system leaders and, hence, may have provided an “elite account,” expressing opinions and perspectives on behalf of their organisation rather than their personal views.^12^ To mitigate this risk we built relationships with quality leads in each of the ICSs, who informed other colleagues of our research well in advance. A second limitation was that we restricted our selection of ICSs to four sites, although as a result we were able to include a wider range of voices per site. Moreover, together, the four ICSs cover a diverse population of around 5 million spanning both rural and urban areas. Hence, the findings from our work can be considered transferable to other ICSs in England and elsewhere.

## Conclusion

 The concept of a single integrated system to address all aspects of health and care for a defined population is compelling. Even though many components remain effectively independent in the re-organised English health and care system, with scope to resist system goals, the role and ambitions of the ICSs offer considerable promise to improve the overall quality and effectiveness of services. However, it is clear that significant time is needed to identify and implement effective interventions and to observe resulting improved outcomes. This will require patience in an overall context where there is considerable political pressure to devote all available energies to more charged and salient problems such as waiting lists.

## Acknowledgements

 The authors would like to acknowledge the support of the local collaborators in each of the participating ICSs. The authors are most grateful for the contributions of Eleni Chambers and Graham Brown, our PPIE colleagues on the Quality, Safety and Outcomes Policy Research Unit. Eleni and Graham provided valuable insights on the qualitative data pertaining to the role of local people, the public and communities in ICSs.

## Ethical issues

 The study received ethical approval from the University of Kent Ethics Committee, reference no LSSJ0459. Researchers approached interview participants by email outlining the purpose of the study and the interview process. Written informed consent was obtained from each participant prior to interview. Participants were assured of confidentiality and anonymity and that participation was voluntary, and that they were free to withdraw from the study. No participants withdrew their consent.

## Conflicts of interest

 Authors declare that they have no conflicts of interest.

## Additional Information

 Co-author Ray Fitzpatrick passed away April 4, 2024.

## References

[1] Garin N, Koyanagi A, Chatterji S (2016). Global multimorbidity patterns: a cross-sectional, population-based, multi-country study. J GerontolA Biol Sci Med Sci.

[2] Heeringa J, Mutti A, Furukawa MF, Lechner A, Maurer KA, Rich E (2020). Horizontal and vertical integration of health care providers: a framework for understanding various provider organizational structures. Int J Integr Care.

[3] Lewis RQ, Checkland K, Durand MA (2021). Integrated care in England - what can we learn from a decade of national pilot programmes?. Int J Integr Care.

[4] Stokes J, Shah V, Goldzahl L, Kristensen SR, Sutton M (2021). Does prevention-focused integration lead to the triple aim? An evaluation of two new care models in England. J Health Serv Res Policy.

[5] NHS England (NHSE). Five Year Forward View. Leeds: NHS England; 2014.

[6] NHS Providers. STPs and Accountable Care: Background Briefing. NHS Providers; 2018.

[7] Baxter S, Johnson M, Chambers D, Sutton A, Goyder E, Booth A (2018). The effects of integrated care: a systematic review of UK and international evidence. BMC Health Serv Res.

[8] NHS England (NHSE). Integrated Care Systems: Design Framework. NHS England; 2021.

[9] Crocker H, Kelly L, Harlock J, Fitzpatrick R, Peters M (2020). Measuring the benefits of the integration of health and social care: qualitative interviews with professional stakeholders and patient representatives. BMC Health Serv Res.

[10] NQB. A Shared for Those Working in Health and Care Systems Developed by the National Quality Board Commitment to Quality. National Quality Board; 2021.

[11] Shah A (2020). How to move beyond quality improvement projects. BMJ.

[12] Green J, Thorogood N. Qualitative Methods for Health Research. SAGE Publications; 2018.

[13] Juran JM. The quality trilogy. In: Wood JC, Wood MC, eds. Joseph M. Juran: Critical Evaluations in Business and Management. Routledge; 2005.

[14] NHS England (NHSE). Core20PLUS5 - An Approach to Reducing Healthcare Inequalities. NHS England; 2022.

[15] Darzi A. High Quality Care for All: NHS Next Stage Review Final Report. London: Department of Health; 2008.

[16] Malley J, Fernández JL (2010). Measuring quality in social care services: theory and practice. Ann Public Coop Econ.

[17] Exworthy M, Blane D, Marmot M (2003). Tackling health inequalities in the United Kingdom: the progress and pitfalls of policy. Health Serv Res.

[18] Ling T, Brereton L, Conklin A, Newbould J, Roland M (2012). Barriers and facilitators to integrating care: experiences from the English Integrated Care Pilots. Int J Integr Care.

[19] Sanderson M, Allen P, Osipovic D, Petsoulas C, Boiko O, Lorne C (2023). Developing architecture of system management in the English NHS: evidence from a qualitative study of three integrated care systems. BMJ Open.

[20] Ostrom E. Governing the Commons: The Evolution of Institutions for Collective Action. Cambridge University Press; 1990.

[21] Waring J, Bishop S, Black G (2023). Navigating the micro-politics of major system change: the implementation of sustainability transformation partnerships in the English health and care system. J Health Serv Res Policy.

[22] Vindrola-Padros C, Ledger J, Hill M, Tomini S, Spencer J, Fulop NJ (2022). The special measures for quality and challenged provider regimes in the English NHS: a rapid evaluation of a national improvement initiative for failing healthcare organisations. Int J Health Policy Manag.

[23] Edwards N, Buckingham H. Strategic Health Authorities and Regions: Lessons from History. Nuffield Trust; 2020.

[24] Kaufman BG, Spivack BS, Stearns SC, Song PH, O’Brien EC (2019). Impact of accountable care organizations on utilization, care, and outcomes: a systematic review. Med Care Res Rev.

[25] Garzón-Orjuela N, Samacá-Samacá DF, Luque Angulo SC, Mendes Abdala CV, Reveiz L, Eslava-Schmalbach J (2020). An overview of reviews on strategies to reduce health inequalities. Int J Equity Health.

[26] Dixon-Woods M, Martin GP (2016). Does quality improvement improve quality?. Future Hosp J.

[27] Lalani M, Bussu S, Marshall M (2020). Understanding integrated care at the frontline using organisational learning theory: a participatory evaluation of multi-professional teams in East London. Soc Sci Med.

[28] Hammond J, Speed E, Allen P, McDermott I, Coleman A, Checkland K (2019). Autonomy, accountability, and ambiguity in arm’s-length meta-governance: the case of NHS England. Public Manag Rev.

